# Serum levels of Wnt5a in Egyptian women with obesity and their association with toll like receptor 2 Arg753Gln gene polymorphism in a pilot case control study of obesity as a state of metaflammation

**DOI:** 10.1038/s41598-025-85470-9

**Published:** 2025-01-21

**Authors:** Shaimaa H. Fouad, Mai Eldeeb, Shereen A. Baioumy, Sara I. Taha, Rehab Ali Ibrahim, Aya Elgendy, Azza Omran, Marwa Hamdy, Raed A. Alharbi, Abdulmajeed A. A. Sindi, Sylvia W. Roman

**Affiliations:** 1 Department of Internal Medicine /Allergy and Clinical Immunology, Ain- Shams University, Cairo, Egypt; 2 Department of Microbiology and Immunology, Faculty of Medicine, Zagazig University, Zagazig, Egypt; 3 Department of Clinical Pathology, Faculty of Medicine, Ain-Shams University, Cairo, Egypt; 4 Department of Physical Medicine/Rheumatology and Rehabilitation, Faculty of Medicine, Ain-Shams University, Cairo, Egypt; 5 Department of Cardiology, Faculty of Medicine, Ain-Shams University, Cairo, Egypt; 6 Department of Medical Biochemistry and Molecular Biology, Ain-Shams University, Cairo, Egypt; 7 Department of Laboratory Medicine, Faculty of Applied Medical Sciences, Al-Baha University, Albaha, Kingdom of Saudi Arabia; 8 Department of Basic Medical Sciences, Faculty of Applied Medical Sciences, Al-Baha University, Albaha, Kingdom of Saudi Arabia; 9 Department of Clinical Pathology, Faculty of Medicine, Ain Shams University, Abbasia, Cairo, 11591 Egypt

**Keywords:** Inflammation, Leptin, Metabolic syndrome, Obesity, TLR2, TNF-α, Wnt5a, Biochemistry, Chemical biology, Immunology

## Abstract

Female obesity is a worldwide health issue linked to chronic metabolic low-grade inflammation (metaflammation) causing multiple obesity-related co-morbid conditions. We aimed to assess the serum levels of wingless integration site family member 5 A (Wnt5a), leptin, and tumor necrosis factor-alpha (TNF-α) as markers of obesity-associated metaflammation and investigate the association with toll-like receptors2 (TLR2) gene (Arg753Gln) single nucleotide polymorphism (SNP) among Egyptian females. The study included 60 females with obesity and 30 matched controls. Serum levels of Wnt5a, leptin, and TNF-α were assessed by ELISA, while TLR2 (Arg753Gln) genotyping was done by PCR-RFLP. Serum Wnt5a, leptin, and TNF-α showed significantly higher levels in females with obesity than controls and a significant increase with higher classes of obesity. They showed significant positive correlations with each other. Only TNF-α and leptin were associated with metabolic syndrome (MetS) among the obesity group. According to TLR2 (Arg753Gln) SNP, the homozygous GG genotype was associated with elevated levels of Wnt5a, leptin, and TNF-α compared to the AA + GA model carriers. No significant differences were found in the distribution of TLR2 Arg753Gln (rs5743708) genotypes and alleles according to obesity or MetS, and the regression analysis showed no significant risk association. Serum Wnt5a, leptin, and TNF-α levels increase in women with obesity and the A allele of TLR2 (Arg753Gln) SNP could be protective against obesity-associated metaflammation.

**Supplementary Information:**

The online version contains supplementary material available at 10.1038/s41598-025-85470-9.

## Introduction

Obesity, a worldwide health burden, is a medical condition with excessive fat storage due to an imbalance between the amount of food consumed and the energy used^1,2^. Women experience obesity more than men as they have less muscle mass, affecting their metabolism and calorie needs^3^.

Excessive fat storage causes the expansion of white adipose tissue with adipocyte hypertrophy that ends with a state of metabolic low-grade chronic inflammation (metaflammation), resulting in obesity-associated metabolic syndrome (MetS), which consists of various cardio-metabolic risk factors resulting in cardiovascular diseases (CVD) and type 2 diabetes (T2DM)^4^. The exact cause that promotes obesity-associated metaflammation is still under research^5^. Several studies pointed to a link between immunological functions and metabolism, as the adipose tissue matrix consists of a compound network of adipocytes and immune cells (most notably, macrophages). Once the activation of adipose tissue macrophage occurs, a cycle of pro-inflammatory cell recruitment cascade takes place^5,6^, leading to an imbalance between the pro-inflammatory and anti-inflammatory adipokines and cytokines, which can explain this metflammation^7–9^.

Tumor necrosis factor (TNF), the first pro-inflammatory cytokine produced in the adipose tissue, acts as a catabolic mediator promoting the production of other pro-inflammatory cytokines, adipokines (such as leptin) and chemokines that cause obesity-associated metaflammation^9^. Leptin is a peptide hormone secreted primarily from the adipose tissue. It is significantly involved in controlling body weight^10^. It binds to hypothalamus receptors to decrease food intake; however, in subjects with obesity, persistent hyperleptinemia causes leptin resistance^11–14^. Adipose tissue-activated macrophages also secrete wingless integration site family member 5 A (Wnt5a) glycoprotein, which induces inflammation via increasing macrophage-related pro-inflammatory cytokines. It was reported that Wnt5a ablation in mice with obesity reduces inflammation in adipose tissue and improves insulin resistance. On the other hand, Wnt5a overexpression increases adipose tissue inflammation and worsens glucose homeostasis^15^.

Furthermore, adipocytes express a broad spectrum of functional toll-like receptors (TLRs) that play a major role in innate immune responses and activate various inflammatory signaling cascades, leading to pro-inflammatory cytokine production and initiation of adaptive immune responses^16^. Of these TLRs is the TLR2, a receptor for lipoproteins, whose signaling cascade relates to obesity and metabolism disorders^17^. It was proved that TLR2 ablation in mice with obesity decreases adipose tissue metaflammation and improves glucose tolerance, promoting TLR2 as a therapy target to prevent subsequent complications^18^. In addition, research reported that the TLR2 gene (Arg753Gln) single nucleotide polymorphism (SNP) is associated with multiple clinical disorders as it negatively influences TLR2 function and attenuates the immune response^19^.

Therefore, the objective of this study was to (1) assess Wnt5a serum levels in association with the well-studied markers, TNF-α and leptin, to gain deeper insights into the mechanisms of obesity-related metaflammation and (2) investigate the link with TLR2 gene (Arg753Gln) SNP in a pilot sample of Egyptian females.

## Methodology

### Ethics

The study was conducted following the ethical guidelines for research defined in the Declaration of Helsinki. The current study protocol has ethical approval number FMASU R215/2022 from Ain Shams University Faculty of Medicine. All participants provided their written informed consent before the start and all data were kept secure and confidential and used exclusively for research.

## Subjects and study settings

This pilot case-control study was conducted in a period from January to November 2023, it included 90 adult females divided into two groups: the group with obesity (*n* = 60) with body mass index (BMI) ≥ 30 Kg/m^2^ and the matched-healthy control group (*n* = 30) with BMI within the normal range. All participants were recruited from the regular attendees of clinics of Ain Shams University Hospitals, Cairo, Egypt. Exclusion criteria included systemic infections, malignancy, pregnancy, and renal, cardiac, and hepatic diseases like viral hepatitis. In addition, participants with other co-morbid conditions and those on oral contraceptive pills or regular medications were also excluded.

### Data collection and clinical assessment

A detailed medical history was obtained from all participants (including age, co-morbidities, chronic diseases, and regularly taken medications) to ensure adherence to the exclusion criteria. Laboratory investigation records of all participants were also collected from their files at the time of blood sampling, including fasting blood sugar (FBS), lipid profile, and erythrocyte sedimentation rate (ESR).

In addition, a clinical examination was done and an assessment of anthropometric measures, including waist circumference, which was measured halfway between the apex of the iliac crest and the bottom edge of the last palpable rib using a stretch-resistant tape; hip circumference, which was measured with a tape parallel to the ground around the broadest part of the buttocks; and weight and height measurement, which was done while participant stood with feet close together and arms at the side.

### Calculations

**Fig. 1:**
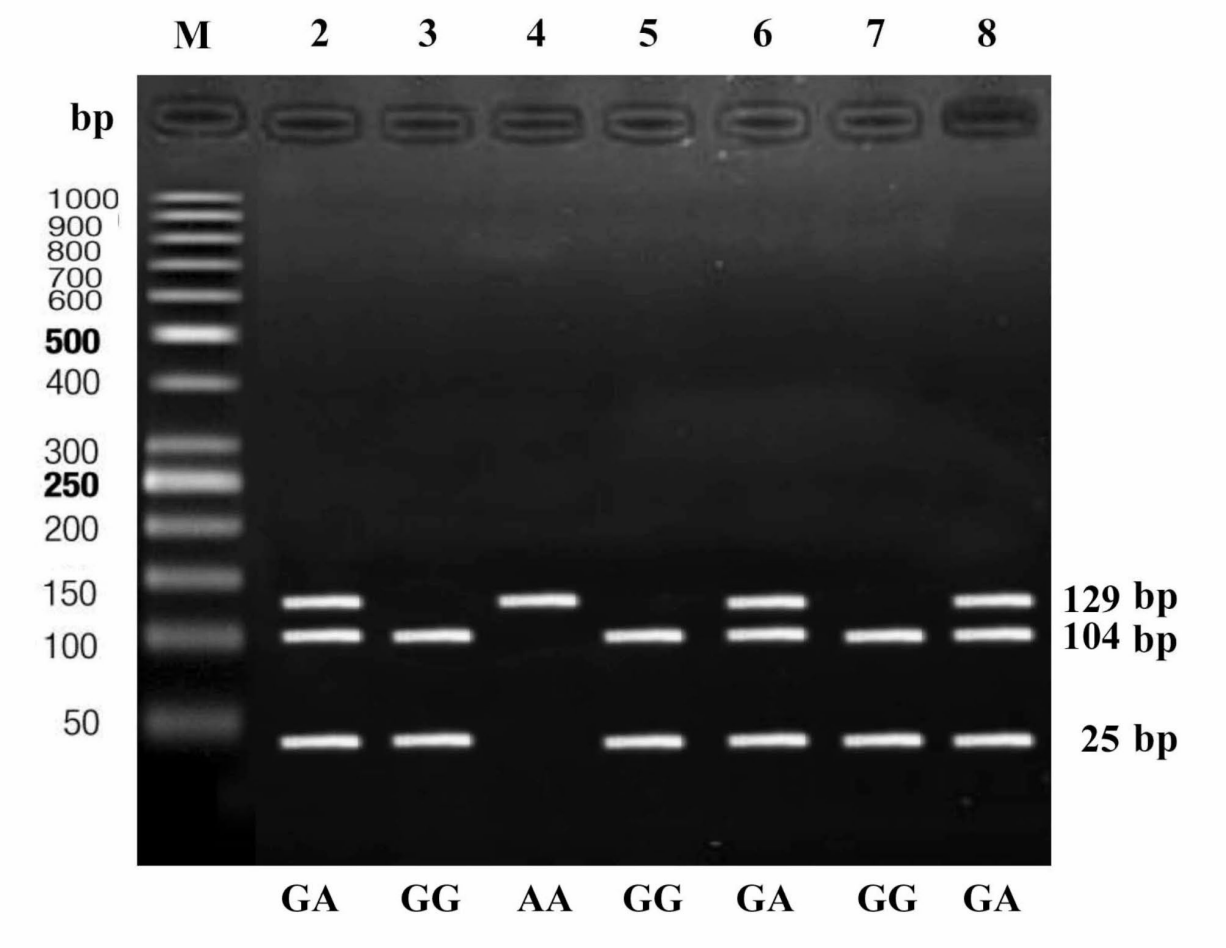
Gel electrophoresis of obesity group samples illustrating different TLR2 (Arg753Gln) genotypes by PCR-RFLP. Lane M: DNA Ladder (1000 bp). The homozygous GG genotype (lanes 3,5,7) of the TLR2 Arg753Gln (rs5743708) showed two bands at 104 bp and 25 bp, while an uncut band indicated the homozygous AA genotype (lane 4) at 129 bp. Three bands at 129,104 bp, and 25 bp indicated the heterozygous GA genotype (lanes 2,6,8).

We calculated the waist/hip ratio (WHR), fat mass index (FMI) (FMI = fat mass (kg) / height (m^2^))^20^, and BMI (BMI = weight (kg)/ height^2^ (m^2^)). Then the patients were categorized into groups according to their obesity class following the CDC classification of obesity^21^. Figure 1.

- Class 1: 40.0% (24/60), BMI 30–34.
- Class 2: 31.7% (19/60), BMI 35–39.
- Class 3: 28.3% (17/60), BMI ≥ 40 .

### Diagnosis of MetS

MetS was diagnosed based on the presence of obesity along with two of the following three criteria: elevated blood pressure, impaired glucose metabolism, and increased non-high-density lipoprotein cholesterol (non-HDL-C) levels (atherogenic dyslipidemia)^22,23^.

### Blood samples collection

In the morning, after an overnight fast (8–12 h), 6 mL of peripheral venous blood was obtained from each participant via a sterile venipuncture divided into two vacutainer tubes: (a) a tube with gel and clot activator in which blood was allowed to clot completely; then, sera were separated by centrifugation and stored at -80°C until analyzed by commercially available human enzyme-linked immunosorbent assay (ELISA) kits for TNF-α, leptin, and Wnt5a. (b) an ethylenediaminetetraacetic acid dipotassium salt (K2-EDTA) tube, which was stored at -80°C until whole blood was used for TLR2 (Arg753Gln) genotyping by polymerase chain reaction-restriction fragment length polymorphism (PCR-RFLP).

### ELISA assessment of metaflammation markers

Samples were diluted according to the recommended dilution guidelines provided by each ELISA kit. Samples that exceeded the detection range of the kit were further diluted for accurate measurement.

- **TNF-α:** (CUSABIO, Houston, TX, USA; Cat. No: CSB-E04740h)

The results were expressed in pg/mL. The kit detection range is 7.8 pg/mL-500 pg/mL with a 1.95 pg/mL sensitivity.

- **Leptin:** (CUSABIO, Houston, TX, USA; Cat. No: CSB-E04649h)

The results were expressed in ng/mL. The kit detection range is 0.156ng/mL-10 ng/mL with a 0.060 ng/mL sensitivity.

- **Wnt5a:** (CUSABIO, Houston, TX, USA; Cat. No: CSB-EL026138HU)

The results were expressed in ng/mL. The kit detection range is 0.156ng/mL-10 ng/mL with a 0.039 ng/mL sensitivity.

### TLR2 (Arg753Gln) genotyping by PCR-RFLP

Each stored EDTA blood sample was used, as whole blood with no prior DNA extraction or sample preparation, in a PCR reaction for TLR2 Arg753Gln (rs5743708) genotyping using Phusion™ Blood Direct PCR kit (pub no. MAN0012900, Thermo Scientific™, USA), according to the manufacturer’s protocol, in a reaction volume of 20µL containing 1 µL (5%) blood, 10µL of 2X Phusion Blood Direct PCR Master Mix, and 0.5 µM of primers^24^ (F:5’dCATTCCCCAGCGCTTCTGCAAGCTCC-3’: R:5’.dGGAACCTAGGACTTTATCGCAGCTC-3’).

In the thermal cycler (Biometra, Germany), PCR cycling conditions were as follows: lysis of cells at 98 ˚ C for 5 min, followed by 40 amplification cycles of denaturation, annealing and extension (at 94 ˚ C for 5 min, at 65 ˚ C for 30 s, and at 72 ˚ C for 30 s, respectively). At last, one cycle of final extension at 72 ˚ C for 10 min, then cooling to 4 ˚ C^25^.

For restriction endonuclease digestion, the resulting PCR product of (129 bp) was treated with Msp1 restriction enzyme (Thermo Scientific™, USA), which recognizes the 5’-CCGG sequence and cleaves between the first and second nucleotides^26^.

Digested products were then loaded on 2% agarose gel and analyzed by electrophoresis. The product was visualized on an ultraviolet transilluminator (Syngene, Frederick, Maryland, USA). Figure 1.

### Statistical analysis

The statistical software utilized for data analysis was IBM SPSS version 21 (IBM Corporation, Armonk, NY, USA). For data comparison, the Chi-square test (χ² test), the Mann–Whitney test, and the Kruskal–Wallis test were used. The correlations were evaluated using the Spearman correlation coefficient. The prediction power of several factors was shown by the area under the curve (AUC) and receiver operating characteristic (ROC). TLR2 (Arg753Gln) genotypes and alleles as independent risk factors for obesity and MetS were identified by regression analysis calculating the odds ratios (ORs) and 95% confidence interval (CI). *P*-values less than 0.05 were designated as significant.

## Results

### Metaflammatory characteristics of the studied groups

This pilot case-control study included 60 females with obesity and 30 matched controls. Their demographic, anthropometric and laboratory characteristics are shown in Table 1.

**Table 1:** Demographic, anthropometric, and laboratory data of the studied groups.

	Groups				Mann-Whitney test	
	Control group (*n* = 30)		Obesity group (*n* = 60)			
	Median	IQR	Median	IQR	Z	*p*-value
Age (years)	43.00	33.00–61.00	46.00	34.50–62.00	0.655	0.512
Weight (Kg)	55.00	50.00–57.00	90.00	83.00–102.50	7.269	< 0.001*
Height (cm)	156.50	150.00–160.00	159.50	154.00–162.00	1.706	0.088
BMI (Kg/m^2^**)**	21.70	20.70–23.60	37.70	32.80–40.25	7.705	< 0.001*
Waist circumference (cm)	89.50	81.00–100.00	112.00	101.50–121.50	5.579	< 0.001*
Hip circumference (cm)	104.00	100.00–112.00	125.00	119.00–132.00	6.509	< 0.001*
WHR	0.82	0.80–0.93	0.90	0.84–0.92	1.886	0.059
FMI (Kg/m^2^**)**	5.45	5.20–6.10	17.15	14.40–19.35	7.707	< 0.001*
FBS (mg/dL)	100.50	90.00–117.00	115.00	104.00–133.50	2.932	0.003*
ESR (mm/hr)	12.00	10.00–13.00	28.00	13.00–41.00	5.394	< 0.001*
Cholesterol (mg/dL)	240.00	156.00–281.00	196.50	186.00–220.50	0.446	0.656
Triglycerides (mg/dL)	138.50	132.00–169.00	150.00	145.00–172.50	2.489	0.013*
HDL-C (mg/dL)	63.00	54.00–79.00	50.00	49.00–64.00	2.549	0.011*
LDL-C (mg/dL)	93.00	81.00–120.00	181.00	147.00–187.00	5.304	< 0.001*
Leptin (ng/mL)	42.50	35.00–50.00	59.75	50.00–73.75	5.644	< 0.001*
TNF-α (pg/mL)	76.00	66.00–89.00	150.00	125.00–350.00	7.546	< 0.001*
Wnt5a (ng/mL)	1.00	0.54–2.30	5.10	4.35–6.68	7.054	< 0.001*

BMI: Body mass index; ESR: Erythrocyte sedimentation rate; FMI: Fat mass index; HDL-C: High density lipoprotein cholesterol; LDL-C: Low density lipoprotein cholesterol; FBS: Fasting blood sugar; TNF-α: Tumor necrosis factor alpha; WHR: Waist-Hip ratio.

*p-value < 0.05 indicates statistical significance.

Regarding the laboratory data and in comparison to the controls, the obesity group showed significantly higher levels of FBS (*p* = 0.003), ESR (*p* < 0.001), triglycerides (TG) (*p* = 0.013), low-density lipoprotein cholesterol (LDL-C) levels (*p* < 0.001), leptin (*p* < 0.001), TNF-α (*p* < 0.001), and Wnt5a (*p* < 0.001). On the other hand, HDL-C levels showed significantly lower levels (*p* = 0.011) among the obesity group compared to the controls. Table 1.

When the obesity group (*n* = 60) was classified according to the obesity class, the metaflammation markers under the study, leptin (*p* < 0.001), TNF-α (*p =* 0.007), and Wnt5a (*p* = 0.001), showed a significant increase with the higher obesity class. Serum leptin and Wnt5a showed significant differences between obesity classes I vs. III (*p-values* < 0.001 and = 0.002, respectively) and II vs. III (*p-values* < 0.001 and 0.006, respectively). On the other hand, the significant difference in serum TNF-α was found only between obesity classes I vs. III (*p* = 0.002). Table 2.

**Table 2:** Characteristics of the 60 females with obesity according to the different obesity class.

	Obesity class						Kruskal-Wallis test		Mann-Whitney test		
	I (*n* = 24)		II (*n* = 19)		III (*n* = 17)						
	Median	IQR	Median	IQR	Median	IQR	X^2^	*P*-value	I&II	I&III	II&III
Age (years)	42.00	34.00–61.00	48.00	38.50–63.50	46.00	36.00–61.00	1.160	0.560	0.281	0.578	0.669
Weight (Kg)	81.00	76.00–86.00	94.00	90.00–101.50	104.00	100.00–110.00	38.788	< 0.001*	< 0.001*	< 0.001*	0.004*
Height (cm)	160.00	153.50–162.50	160.00	155.00–	158.00	153.00–160.00	0.705	0.703	0.777	0.465	0.483
BMI (Kg/m^**2**^**)**	32.00	31.00–33.00	37.90	36.60–39.00	42.20	41.00–43.50	52.065	< 0.001*	< 0.001*	< 0.001*	< 0.001*
Waist circumference (cm)	101.00	97.50–110.50	116.00	109.50–119.50	124.00	120.00–128.00	28.710	< 0.001*	0.001*	< 0.001*	0.004*
Hip circumference (cm)	119.50	113.50–123.00	127.00	122.00–132.00	132.00	130.00–135.00	27.200	< 0.001*	0.001*	< 0.001*	0.013*
WHR	0.88	0.82–0.91	0.90	0.82–0.91	0.90	0.89–0.94	3.737	0.154	0.460	0.058	0.401
FMI (Kg/m^**2**^**)**	14.25	13.00–15.75	17.40	17.00–19.00	19.50	18.10–20.30	30.782	< 0.001*	< 0.001*	< 0.001*	0.003*
FBS (mg/dL)	110.00	103.00–133.50	115.00	107.50–136.50	119.00	102.00–122.00	0.847	0.655	0.316	0.853	0.657
ESR (mm/hr)	20.00	13.00–32.50	30.00	16.00–45.00	30.00	25.00–55.00	3.201	0.202	0.269	0.071	0.690
Cholesterol (mg/dL)	186.00	154.50–202.50	193.00	186.00–220.00	221.00	215.00–287.00	9.987	0.007*	0.100	0.003*	0.058
Triglycerides (mg/dL)	145.00	145.00–150.00	155.00	144.50–166.50	167.00	156.00–179.00	10.560	0.005*	0.443	0.001*	0.027*
HDL-C (mg/dL)	50.00	49.00–64.00	49.00	48.00–62.00	51.00	45.00–66.00	0.361	0.835	0.586	0.651	0.836
LDL-C (mg/dL)	157.50	147.00–182.00	162.00	147.00–190.00	183.00	166.00–184.00	2.587	0.274	0.496	0.082	0.544
Leptin (ng/mL)	52.50	44.00–55.50	60.50	55.00–65.10	85.00	66.00–87.50	24.868	< 0.001*	0.093	< 0.001*	< 0.001*
TNF-α (pg/mL)	130.00	121.00–177.50	160.00	125.00–350.00	390.00	150.00–650.00	9.959	0.007*	0.205	0.002*	0.063
Wnt5a (ng/mL)	4.65	3.98–5.25	5.40	4.65–5.95	7.60	6.50–8.03	13.042	0.001*	0.084	0.002*	0.006*

BMI: Body mass index; ESR: Erythrocyte sedimentation rate; FMI: Fat mass index; HDL-C: High density lipoprotein cholesterol; LDL-C: Low density lipoprotein cholesterol; FBS: Fasting blood sugar; TNF-α: Tumor necrosis factor alpha; WHR: Waist-Hip ratio.

*p-value < 0.05indicates statistical significance.

The metaflammation markers (leptin, TNF-α, and Wnt5a) showed significant positive correlations with each other (*p* < 0.001) and with body weight, waist and hip circumferences, FMI, BMI, ESR, and TG. On the other hand, leptin and Wnt5a showed a positive correlation with cholesterol and LDL-C levels. And only TNF-α showed a significant negative correlation with HDL-C (*p* = 0.009). Table 3.

**Table 3:** Correlations of metaflammation markers (leptin, Wnt5a and TNF-α) with the demographic, anthropometric and laboratory data among all participants.

	Leptin		Wnt5a		TNF-α	
	*r*	*P*-value	*r*	*P*-value	*r*	*P*-value
Leptin (ng/mL)	--	--	0.609	< 0.001^*^	0.556	< 0.001^*^
TNF-α (pg/mL)	0.556	< 0.001^*^	0.492	< 0.001^*^	--	--
Wnt5a (ng/mL)	0.609	< 0.001^*^	--	--	0.492	< 0.001^*^
Age (years)	-0.053	0.686	0.164	0.210	-0.145	0.270
Weight (Kg)	0.480	< 0.001^*^	0.327	0.011^*^	0.462	< 0.001^*^
Height (cm)	-0.164	0.211	-0.117	0.374	0.150	0.254
BMI (Kg/m^2^**)**	0.635	< 0.001^*^	0.500	< 0.001^*^	0.462	< 0.001^*^
Waist circumference (cm)	0.411	0.001^*^	0.447	< 0.001^*^	0.326	0.011^*^
Hip circumference (cm)	0.492	< 0.001^*^	0.415	0.001^*^	0.370	0.004^*^
WHR	0.110	0.402	0.193	0.140	0.057	0.666
FMI (Kg/m^2^**)**	0.514	< 0.001^*^	0.470	< 0.001^*^	0.299	0.020^*^
FBS (mg/dL)	-0.030	0.823	0.085	0.518	0.057	0.668
ESR (mm/hr)	0.390	0.002^*^	0.499	< 0.001^*^	0.337	0.008^*^
Cholesterol (mg/dL)	0.495	< 0.001^*^	0.353	0.006^*^	0.153	0.244
Triglycerides (mg/dL)	0.507	< 0.001^*^	0.390	0.002^*^	0.582	< 0.001^*^
HDL-C (mg/dL)	-0.052	0.693	0.093	0.481	-0.332	0.009^*^
LDL-C (mg/dL)	0.308	0.016^*^	0.452	< 0.001^*^	0.242	0.062

BMI: Body mass index; ESR: Erythrocyte sedimentation rate; FMI: Fat mass index; HDL-C: High density lipoprotein cholesterol; LDL-C: Low density lipoprotein cholesterol; FBS: Fasting blood sugar; TNF-α: Tumor necrosis factor alpha; WHR: Waist-Hip ratio.

*p-value < 0.05 indicates statistical significance.

Among the obesity group, 51.67% (31/60) fulfilled the criteria of MetS. Levels of serum leptin and TNF-α showed a significant increase (*p-values =* 0.012 and 0.005, respectively) in participants with MetS compared to those without. However, levels of serum Wnt5a showed a non-significant difference (*p* = 0.061). Table 4.

**Table 4:** Comparison of the characteristics of the obesity group (*n* = 60) according to the presence of metabolic syndrome.

Obese	Metabolic syndrome				Mann-Whitney test	
	No (*n* = 29)		Yes (*n* = 31)			
	Median	IQR	Median	IQR	Z	*P*-value
Age (years)	44.00	33.00–54.00	47.00	36.00–62.50	1.125	0.261
Weight (Kg)	88.00	80.00–95.00	97.50	85.00–103.50	1.749	0.080
Height (cm)	160.00	155.00–163.00	159.00	153.00–160.50	1.092	0.275
BMI (Kg/m^2^**)**	33.50	31.20–38.50	39.00	34.50–41.70	2.619	0.009*
Waist circumference (cm)	108.00	100.00–119.00	118.00	110.00–125.50	2.139	0.032*
Hip circumference (cm)	121.00	117.00–128.00	127.00	122.00–132.50	2.088	0.037*
WHR	0.89	0.82–0.92	0.90	0.86–0.91	0.328	0.743
FMI (Kg/m^2^**)**	16.00	13.60–17.40	18.00	16.65–20.00	2.598	0.009*
FBS (mg/dL)	110.00	101.00–133.00	117.00	108.50–131.00	1.206	0.228
ESR (mm/hr)	22.00	12.00–30.00	30.00	20.00–60.00	2.168	0.030*
Cholesterol (mg/dL)	186.00	180.00–193.00	220.00	193.00–277.00	2.761	0.006*
Triglycerides (mg/dL)	145.00	140.00–145.00	166.00	156.00–178.00	5.449	< 0.001*
HDL-C (mg/dL)	50.00	49.00–64.00	49.00	45.00–66.50	1.528	0.127
LDL-C (mg/dL)	147.00	147.00–181.00	183.00	173.50–190.00	3.253	0.001*
Leptin (ng/mL)	55.00	46.00–65.00	65.00	55.00–80.25	2.512	0.012*
TNF-α (pg/mL)	130.00	125.00–150.00	350.00	137.50–445.00	2.777	0.005*
Wnt5a (ng/mL)	4.70	4.00–6.00	5.50	4.70–7.55	1.872	0.061

BMI: Body mass index; ESR: Erythrocyte sedimentation rate; FMI: Fat mass index; HDL-C: High density lipoprotein cholesterol; LDL-C: Low density lipoprotein cholesterol; FBS: Fasting blood sugar; TNF-α: Tumor necrosis factor alpha; WHR: Waist-Hip ratio.

*p-value < 0.05 indicates statistical significance.

**Fig. 2:**
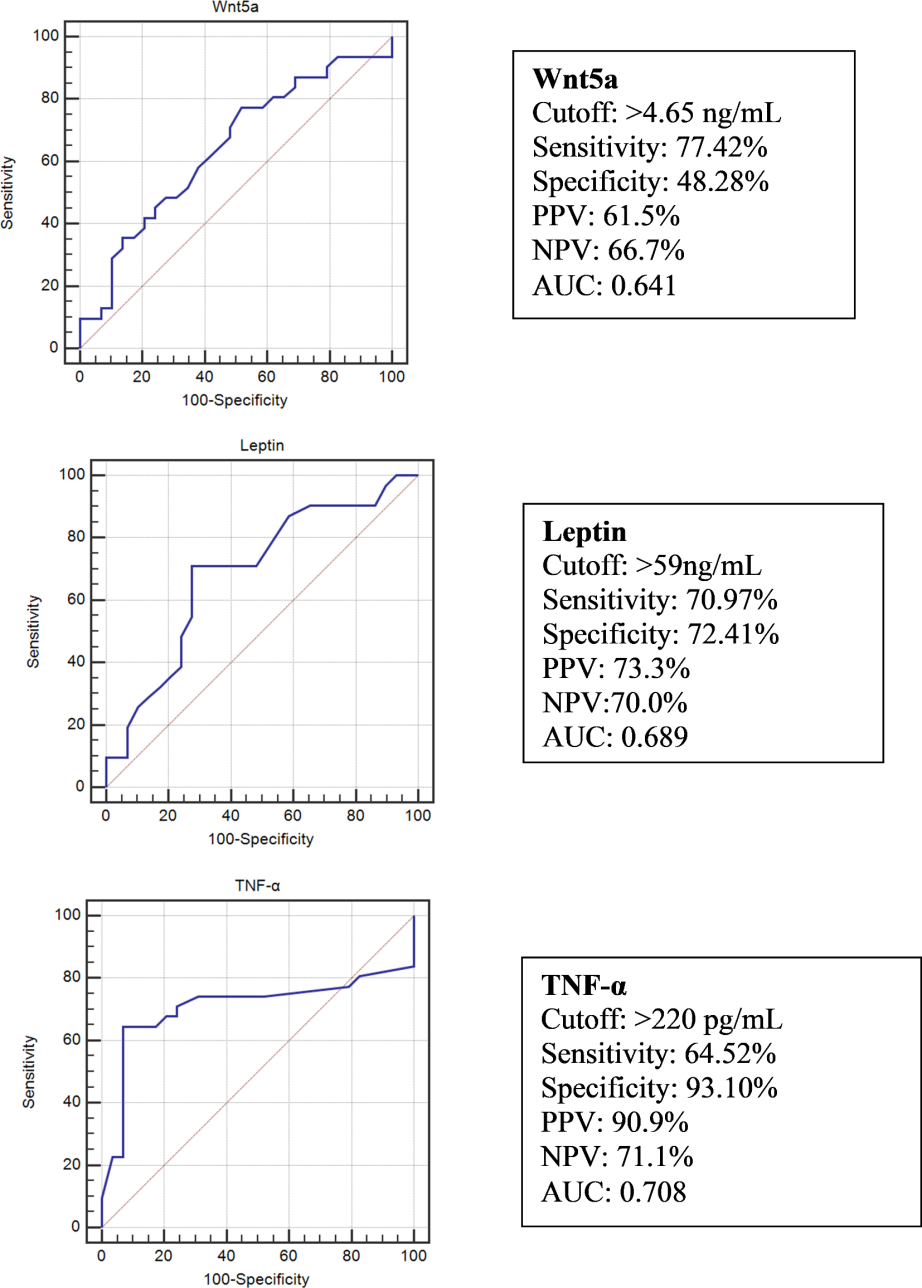
ROC curve analyses of Wnt5a, leptin, and TNF-α among the obesity group (*n* = 60) for metabolic syndrome predictive ability.

ROC curve analyses were established to assess the ability of the metaflammation markers (leptin, TNF-α, and Wnt5a) to predict MetS. The AUC values, ranked from highest to lowest, were as follows: TNF-α at 0.708, leptin at 0.689, and Wnt5a at 0.641. Figure 2.

### **TLR2 (Arg753Gln) SNP and metaflammatory characteristics**

There were no significant differences in the distribution of TLR2 (Arg753Gln) genotypes (*p* = 0.691) and alleles (*p* = 0.415) between the obesity and control groups, with the homozygous GG genotype and the G allele being the most prevalent in both groups. Only one case (1.67%) in the obesity group had the homozygous AA genotype, and no one in the control group had this genotype. The frequency of the heterozygous GA genotype was 13.33% (8/60) in the obesity group and 10% (3/30) in the control group. The OR (95% CI) for the AA and GA genotypes and the A allele did not reach statistical significance (*p-values* > 0.05). Table 5.

**Table 5:** Distribution of TLR2 (Arg753Gln) genotypes and alleles among the obesity group (*n* = 60) versus controls (*n* = 30) and according to metabolic syndrome among the obesity group (*n* = 60).

TLR2 (Arg753Gln)		Groups				Chi-square		Odd’s ratio	95% CI	*p*-value
		Control group (*n* = 30)		Obesity group (*n* = 60)						
		n	%	n	%	X^2^	*p*-value			
Genotypes	AA	0	0.00	1	1.67	0.740	0.691	0.624	0.025–15.843	0.775
	GA	3	10.00	8	13.33			0.708	0.174–2.891	0.631
	GG	27	90.00	51	85.00			-	-	-
Alleles	A	3	5.00	10	8.33	0.663	0.415	0.579	0.153–2.188	0.420
	G	57	95.00	110	91.67			-	-	-

TLR2 (Arg753Gln)		Metabolic syndrome				Chi-square		Odd’s ratio	95% CI	*p*-value
		No ( n = 29)		Yes (n = 31)						
		n	%	n	%	X^**2**^	*p*-value			
Genotypes	AA	1	3.45	0	0.00	1.926	0.382	3.638	0.142–93.545	0.436
	GA	5	17.24	3	9.68			2.029	0.438–9.408	0.366
	GG	23	79.31	28	90.32			–	–	–
Alleles	A	7	12.07	3	4.84	2.051	0.152	2.699	0.663–10.985	0.166
	G	51	87.93	59	95.16			–	–	–

*p*-value < 0.05 indicates statistical significance.

In addition, there were no significant differences in the distribution of TLR2 (Arg753Gln) genotypes (*p* = 0.382) and alleles (*p* = 0.152) in the obesity group when compared according to MetS, with the homozygous GG genotype and the G allele being the most prevalent in both groups. Only one case (3.45%) in the obesity group with no MteS had the homozygous AA genotype, and no one in the obesity group with MteS had this genotype. The frequency of the heterozygous GA genotype was 17.24% (5/29) in the obesity group with no MteS and 9.68% (3/31) in the obesity group with MteS. The OR (95% CI) for the AA and GA genotypes and the A allele did not reach statistical significance (*p-values* > 0.05). Table 5.

When characteristics of the obesity group were compared according to TLR2 (Arg753Gln) genotypes, the BMI (*p =* 0.019), waist circumference (*p* = 0.031), hip circumference (*p =* 0.012), FBS (*p* = 0.023), cholesterol (*p* = 0.041), LDL-C (*p* = 0.043), leptin (*p* = 0.018), TNF-α (*p =* 0.025), and Wnt5a (*p* < 0.001) showed significantly lower values in the AA + GA model carriers compared to the homozygous GG genotype group. Table 6.

**Table 6:** Comparison of the obesity group (*n* = 60) according to TLR2 (Arg753Gln) genotypes.

Obesity group (*n* = 60)	TLR2 (Arg753Gln) gene polymorphism				Mann-Whitney test	
	AA + GA		GG			
	Median	IQR	Median	IQR	Z	*p*-value
Age (years)	47.00	37.00–48.00	46.00	34.00–62.00	0.041	0.967
Weight (Kg)	84.00	75.00–90.00	90.00	84.75–103.00	1.898	0.058
Height (cm)	158.00	155.00–160.00	160.00	153.50–162.00	0.291	0.771
BMI (Kg/m^2^**)**	31.20	30.70–35.50	37.80	33.00–40.65	2.351	0.019*
Waist circumference (cm)	100.00	98.00–115.00	116.00	107.00–123.00	2.155	0.031*
Hip circumference (cm)	117.00	114.00–125.00	126.00	120.00–132.00	2.508	0.012*
WHR	0.90	0.84–0.90	0.90	0.84–0.92	0.104	0.917
FMI (Kg/m^2^**)**	15.00	13.00–16.00	17.40	14.75–19.45	1.927	0.054
FBS (mg/dL)	100.00	87.00–109.00	116.00	107.50–135.00	2.278	0.023*
ESR (mm/hr)	20.00	13.00–28.00	30.00	13.00–55.00	1.320	0.187
Cholesterol (mg/dL)	186.00	154.00–186.00	205.00	186.00–226.00	2.042	0.041*
Triglycerides (mg/dL)	145.00	145.00–145.00	155.00	145.00–178.00	1.574	0.116
HDL-C (mg/dL)	49.00	49.00–54.00	50.00	48.00–65.00	0.438	0.661
LDL-C (mg/dL)	147.00	147.00–165.00	181.00	147.00–190.00	2.026	0.043*
Leptin (ng/mL)	50.00	42.50–55.50	60.50	55.00–75.25	2.375	0.018*
TNF-α (pg/mL)	125.00	122.00–130.00	180.00	125.00–405.00	2.244	0.025*
Wnt5a (ng/mL)	3.50	3.10–3.70	5.50	4.70–7.00	4.484	< 0.001*

BMI: Body mass index; ESR: Erythrocyte sedimentation rate; FMI: Fat mass index; HDL-C: High density lipoprotein cholesterol; LDL-C: Low density lipoprotein cholesterol; FBS: Fasting blood sugar; TNF-α: Tumor necrosis factor alpha; WHR: Waist-Hip ratio.

*p-value < 0.05 indicates statistical significance.

## Discussion

### Obesity as a state of metaflammation

The tremendous effects of obesity on women’s health are considered catastrophic. The incidence of MetS, T2DM, polycystic ovarian disease, and some cancers significantly increase with obesity^3^. The metabolic and immune systems are considered essential for human survival. The regulation of metabolism and immune responses occurs through highly combined processes. Now, the adipose tissue is pointed to as a metabolically active organ of particular importance. In people with obesity, it is considered as a source of metaflammatin due to the aberrant release of cytokines and activation of inflammatory signalling pathways. Such chronic metaflammation process precedes the onset of complications^27,28^.

The three markers under the current study (Wnt5a, leptin, and TNF-α) showed significantly higher levels among obesity groups than controls, and their higher levels were associated with higher obesity class. They were also positively correlated with each other. Notably, only TNF-α and leptin were associated with MetS among the obesity group.

Hotamisligil and coworkers neutralized serum TNF-α in obese rats to study its catabolic effect and found an increase in the peripheral utilization of glucose in reaction to insulin. Their results and ours indicate a role for TNF-α in insulin resistance and MetS accompanying obesity^29^. In contrast, A meta-analysis concluded that TNF-α blockers in treating many disorders were linked to weight gain and increased BMI as well-known side effects. Therefore, targeting the immune system and modulating the signaling of the different cytokines can be considered a new pharmacological approach to treating many eating disorders, including anorexia nervosa and cancer cachexia^30^.

Furthermore, TNF-α was the first proinflammatory cytokine linked to insulin resistance and T2DM pathogenesis as it decreased the expression of insulin-regulated glucose transporter type 4 (GLUT4), found mainly in adipocytes. Moreover, TNF-α inhibited peripheral insulin action, leading to insulin resistance via the serine phosphorylation of insulin receptor substrate-1 (IRS-1)^31–33^.

Leptin plays many vital roles, such as regulating food intake, immune responses, and body weight. Its effects are mediated via its receptor, distributed in the central nervous system and other tissues, such as adipocytes^34^.

A review article by Obradovic et al. reported two significant characteristics of typical obesity: hyperleptinemia and resistance to reducing body mass. They reported that leptin gene level overexpression was found in the adipose tissue of individuals with obesity and had a strong positive correlation with body fat percentage. Additionally, under conditions of food excess, many neurological dysfunctions have been linked to high leptin levels^11^.

In addition, Lozano-Bartolomé et al. reported that elevated levels of leptin and TNF-α in obesity activate neutrophils, originating peripherally, not from fat-resident cells, to infiltrate the visceral adipose tissue and start adipose tissue inflammation. Leptin results in neutrophil activation via indirect production of TNF-α by monocytes^35^.

Also, in line with our results, Koutaki et al. reported that Wnt5a expression increased proportionally to obesity in rodent model experimental results. It acts by activating c-Jun N-terminal kinase 1 (JNK1) which blocks the activity of IRS-1 in adipocytes, leading to decreased insulin signaling and the enhancement of insulin resistance^36^. Insulin resistance encourages the storage of fat in the abdominal area, especially around visceral organs. This pattern of fat distribution is strongly linked to MetS^37^. Koutaki et al. also reported that Wnt5a is linked to oxidative stress through the enhancement of nicotinamide adenine dinucleotide phosphate (NADPH) oxidase activity, linking inflammation to metabolism^36^.

Prats-Puig et al. measured levels of Wnt5a in 342 prepubertal children to assess their link to metabolic markers. Also, they studied Wnt5a in conditioned media of adipose tissue explants collected from 12 children. The results showed a positive correlation between serum and conditioned media and metabolic dysfunction^8^.

In obesity, Wnt5a promote adipogenesis and fat deposition in adipose cells via noncanonical Wnt signalling and inhibiting the canonical Wnt pathway. The non-canonical signaling pathway is primarily associated with insulin resistance and inflammation of the endothelium^36,38,39^. SFRP5 is an anti-inflammatory adipocytokine that binds to Wnt5a, blocking its receptor binding and thereby counteracting Wnt5a-induced inflammatory responses. Artemniak-Wojtowicz et al. observed that the Wnt5a/SFRP5 ratio rises with increasing obesity^40^.

### Association with TLR2 (Arg753Gln) SNP

TLR2 has been linked to obesity and MetS. Free fatty acids in the diet induce the secretion of matrix metalloproteinase 3, which activates TLR2 signaling^41^. Also, the increased circulating lipoproteins in obesity stimulate TLR2, causing adipocytes and macrophages to secrete less adiponectin and to produce pro-inflammatory cytokines, predisposing to leptin resistance in the central nervous system and insulin resistance peripherally^42,43^. Caricilli et al. reported that inhibition of TLR2 improved insulin sensitivity by decreasing local inflammatory cytokine expression in adipose tissue and muscles of obese mice^44^. Furthermore, Himes and Smith found that TLR2-deficient mice showed reduced adipocyte hypertrophy, macrophage infiltration, and production of inflammatory cytokines in adipose tissue, as well as protection against diet-induced obesity and insulin resistance^45^. This finding was also supported by other studies^46,47^.

Obesity is recognized to be the outcome of a complicated interplay between environmental and genetic variables. Disruption or mutations of TLRs in experimental models were protective from obesity-associated insulin resistance^41^, and TLR2 Arg753Gln (rs5743708) SNP was associated with multiple clinical disorders^48,49^.

The current study found no statistically significant differences in genotypes or alleles distribution of TLR2 Arg753Gln (rs5743708) SNP between obesity and control groups and within the obesity group according to the presence or absence of MetS. However, the AA + AG model carriers showed more favorable metabolic phenotypes and lower metaflammation markers. This finding highlights the possibility that the A allele may be protective against the obesity-associated metaflammation. Larger-scale studies which include both sexes are recommended to prove this association.

Our findings could be explained by what was published by Tyurin et al., who studied TLR2 (Arg753Gln) SNP in atopic dermatitis, that patients with the heterozygous GA genotype had higher serum levels of T helper-2 interleukins (IL-4 and IL-10) compared to controls and patients with the homozygous GG genotype. Furthermore, IL-10 is an antagonist to many cytokines; it inhibits the release of TNF, IL-6, and IL-1β by monocytes^49^. According to Vuononvirta et al., the TLR2 (Arg753Gln) SNP interferes with cell activation processes by blocking the TLR2 signaling pathway^50^.

Despite the current study being the first to assess the link between TLR2 (Arg753Gln) SNP and obesity metaflammation, it was not free from limitations that included the relatively small sample size that was disproportionate between the obesity group and controls. It is recommended to involve a larger sample size with proper sample size calculation due to the infrequency of TLR2 (Arg753Gln) SNP and to assess genes of other members of the TLRs, mainly TLR4, the primary receptor for free fatty acids.

In conclusion, this study identified three markers, Wnt5a, leptin, and TNF-α which were significantly higher in women with obesity and positively correlated with each other. Moreover, the AA + AG model carriers of TLR2 (Arg753Gln) SNP had more favorable metabolic profiles and lower levels of metaflammation markers.

## Electronic supplementary material

Below is the link to the electronic supplementary material.

Supplementary Material 1

Supplementary Material 2

Supplementary Material 3

Supplementary Material 4

Supplementary Material 5

Supplementary Material 6

## Data Availability

On reasonable request, the corresponding author will provide the datasets used and/or analyzed during the current work.
