# Supplementary figures and images for: Serum levels of Wnt5a in Egyptian women with obesity and their association with toll like receptor 2 Arg753Gln gene polymorphism in a pilot case control study of obesity as a state of metaflammation
Source: Sci Rep. 2025 Jan 21;15:2702. doi: 10.1038/s41598-025-85470-9 (PMC11751074; doi:10.1038/s41598-025-85470-9)

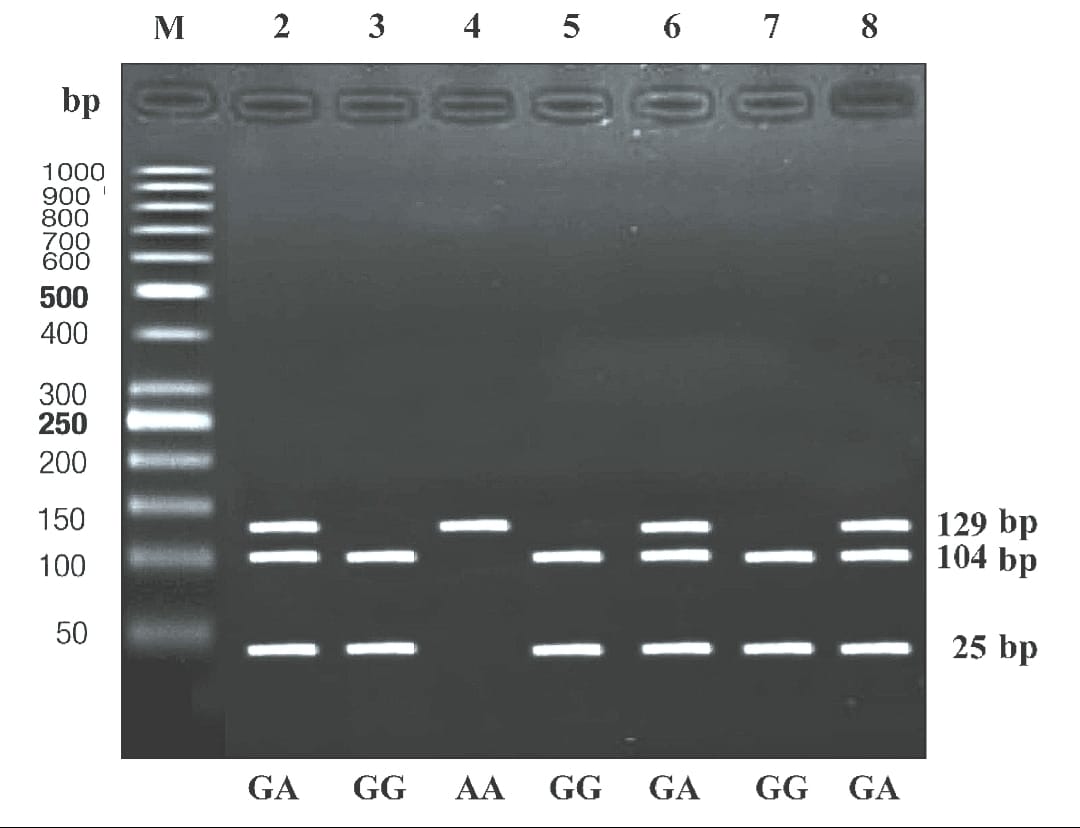

Supplement: Supplementary file 1 — Supplementary Material 1 [file 41598_2025_85470_MOESM1_ESM.jpeg]

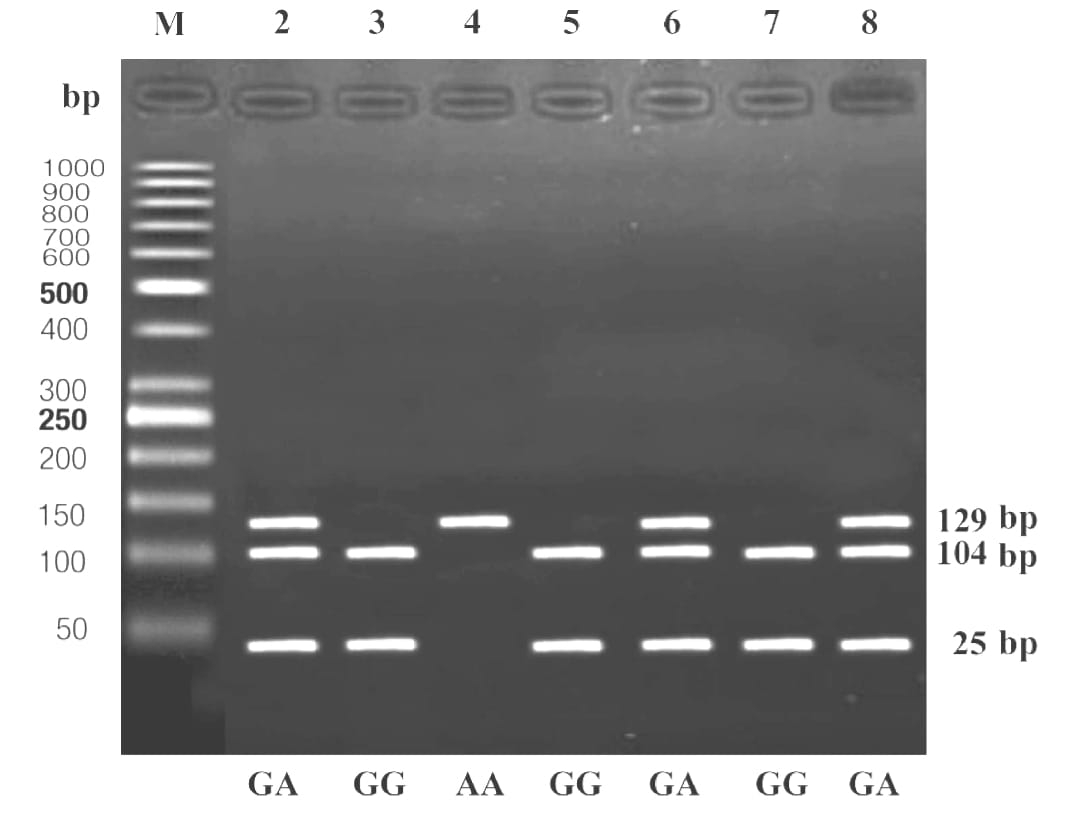

Supplement: Supplementary file 2 — Supplementary Material 2 [file 41598_2025_85470_MOESM2_ESM.jpeg]

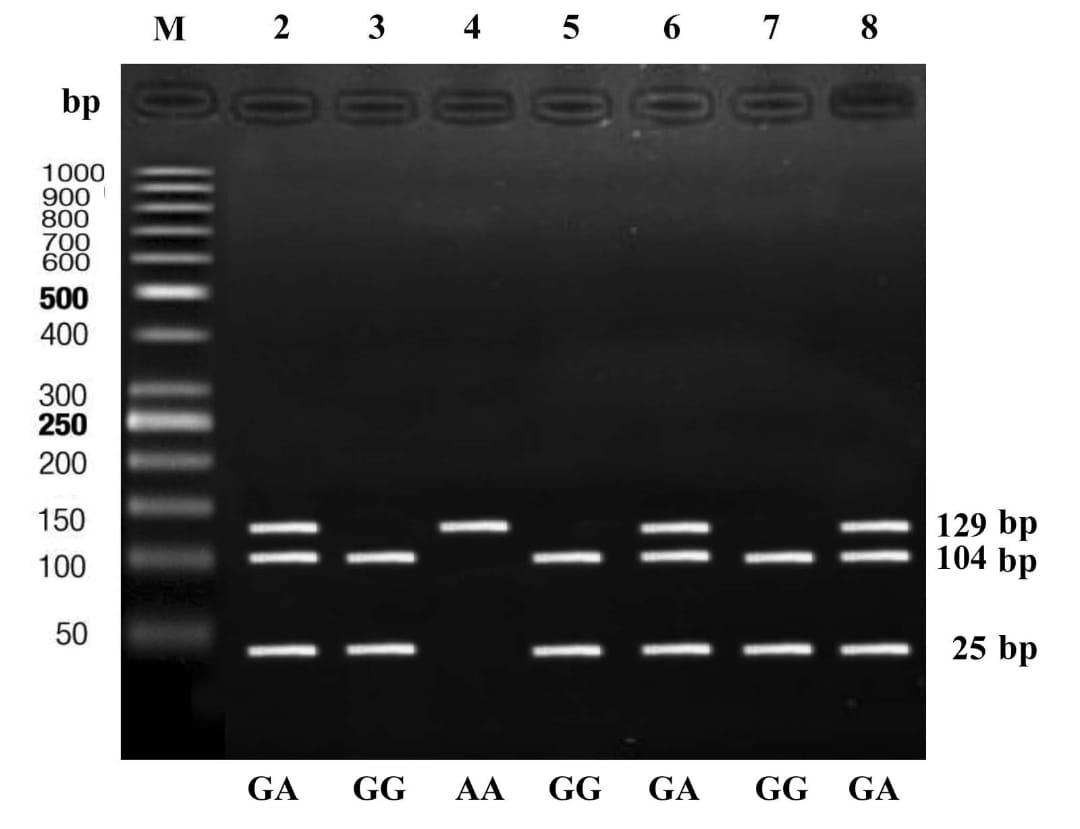

Supplement: Supplementary file 3 — Supplementary Material 3 [file 41598_2025_85470_MOESM3_ESM.jpeg]

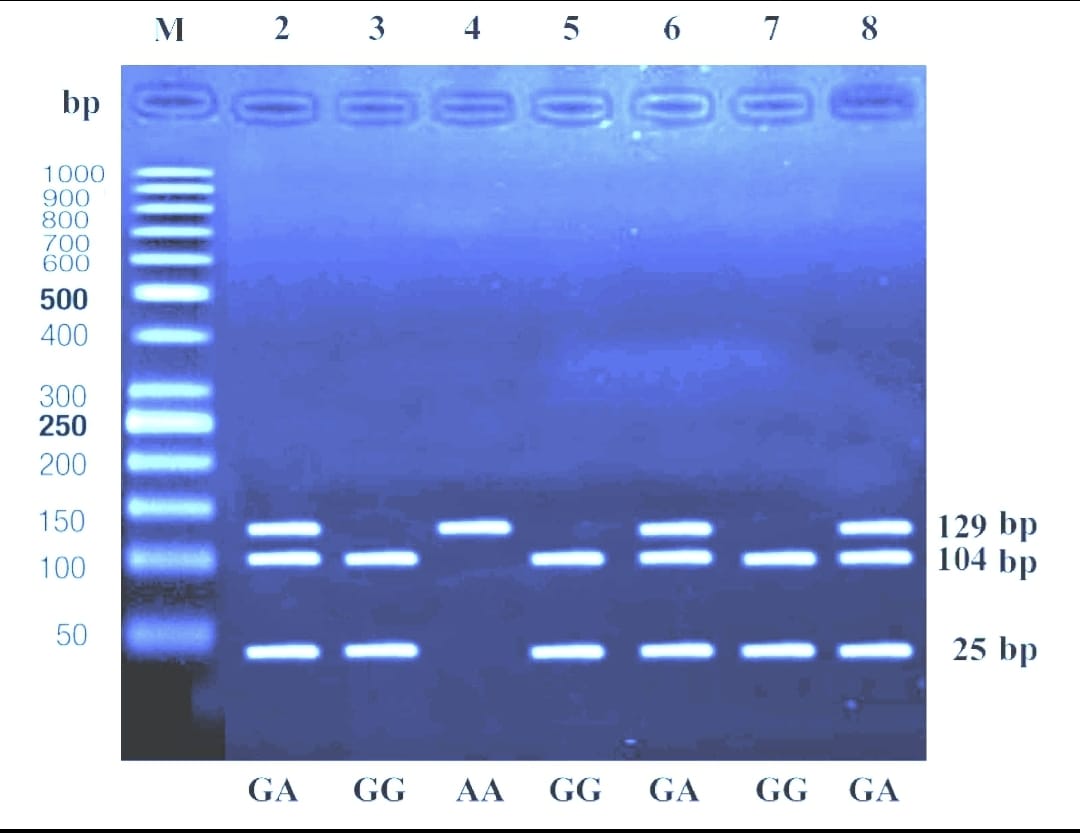

Supplement: Supplementary file 4 — Supplementary Material 4 [file 41598_2025_85470_MOESM4_ESM.jpeg]

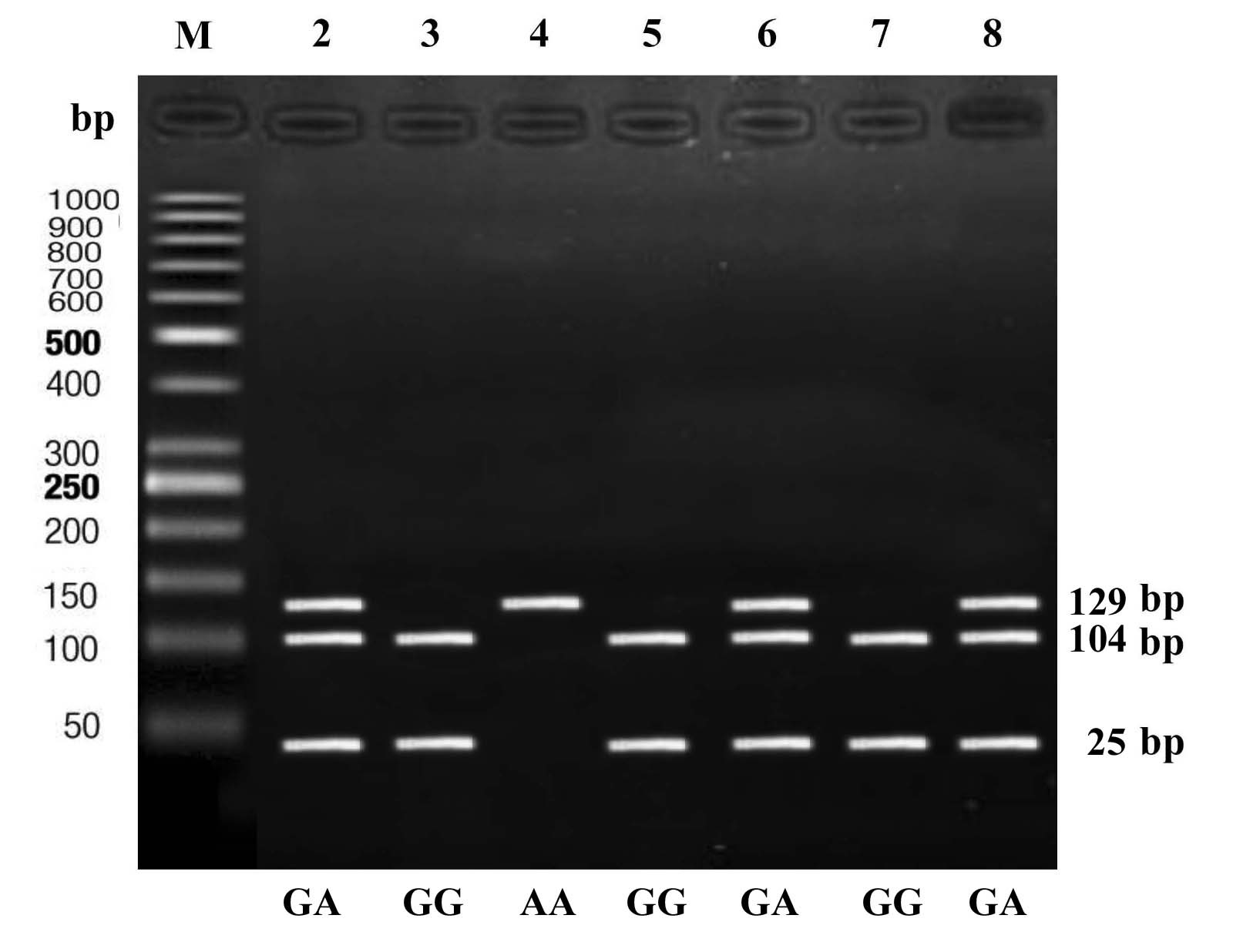

Supplement: Supplementary file 5 — Supplementary Material 5 [file 41598_2025_85470_MOESM5_ESM.jpeg]
